# Study on the anti-interference characteristics of neuronal networks: a comparative study of chemical synapses and electrical synapse

**DOI:** 10.3389/fnins.2025.1581347

**Published:** 2025-07-03

**Authors:** Xiang Li, Mai Lu

**Affiliations:** Key Laboratory of Opto-Electronic Technology and Intelligent Control, Ministry of Education, Lanzhou Jiaotong University, Lanzhou, Gansu, China

**Keywords:** HH neuron model, neuro synapses, anti-interference, simulation research, correlation coefficient

## Abstract

The synapses and network topology enhance neural synchronization and anti-interference, enabling the bio-inspired brain model to mimic biological noise resilience effectively. This study numerically simulates the effects of synapses and network topology on the synchronous discharge and anti-interference of neuronal networks. The Hodgkin–Huxley neuron model, the electrical synapses (ES), the Hansel chemical synapse (HS), and the Rabinovich chemical synapse (RS) were used to construct the neural networks with the ring structure and the Newman–Watts (NW) small-world topology. The sine wave and the sine wave with superimposed Gaussian white noise interference were selected as the stimulation signals. The MATLAB and Simulink platforms were employed to implement the numerical simulation. For the ring network with the sine wave stimulation, the correlation coefficients of one set of neuron pairs (neuron 1 and neuron 25) were 0.292 (ES), 0.236 (HS), and 0.168 (RS), respectively. However, after superimposed interference, the correlation coefficients become 0.099, 0.086, and 0.379, respectively. For the NW small-world topology with sinusoidal stimulation, the correlation coefficients of the same neuron pair were 0.569 (ES), 0.563 (HS), and 0.969 (RS), respectively. The correlation coefficients after superposition interference become 0.569, 0.163, and 0.88, respectively. The HS-coupled network exhibits severe signal latency (Ring network: Latency >200 ms, NW small-world network: Latency >150 ms). While RS-coupled network demonstrates dramatically reduced delays (<50 ms) across both topologies. The results suggest that the synchronization of the RS coupling network is much better than that of both ES and HS coupling networks. Ring networks coupled via HS demonstrate performance metrics comparable to those of ES-coupled ring networks, albeit with significant action potential propagation delays observed in both configurations. The NW small-world network can reduce the delay of signal transmission in the network by increasing the number of pathways. As network topological complexity increases, distal neurons demonstrate reduced spike timing variability and enhanced firing synchrony, collectively improving interference suppression efficacy.

## 1 Introduction

As an extremely sophisticated and perfect information system, the brain is the command center of human life activities. Neurons are the most basic structural and functional units of the nervous system, and synapses are the key structures that support information transmission between neurons, playing a major role in the realization of brain function (Pereda, 2014; Magee and Grienberger, 2020). The nervous system needs to complete the transmission, integration, and coordination of bioelectrical signals and process a large amount of information. There is noise interference in the process of processing information in the nervous system. The known interference sources include the internal environmental noise generated by the ion exchange process and the external input noise. The interference will change the neuron discharge (Stevens and Zador, 1998; Destexhe and Rudolph-Lilith, 2012; Lindner, 2004; Faisal et al., 2008). However, the nervous system can complete the information processing work quite accurately in the presence of interference, which depends on the anti-interference ability of the nervous system. Therefore, the anti-interference mechanism and ability of the nervous system are the basis for the normal operation of the nervous system. A large number of studies have shown that some mechanisms and characteristics of synapses and network topologies have the ability to affect the synchronous discharge and anti-interference of neuronal networks. Beggs and Plenz (2003) revealed that the brain can dynamically balance information processing needs and network stability through critical states. O'Byrne and Jerbi (2022) studied the correlation between the critical state and the neural model and found that the feedforward network model under the critical state is superior to the traditional model in noise robustness and task adaptability, especially in dealing with time-dependent tasks. Das and Levina (2019) proposed that the neural network of a healthy brain maintains a critical state through a self-organizing mechanism. Studies in the field of neuroscience have shown that the brain can achieve perfect synchronous discharge and anti-interference functions by self-adjusting segments to reach a critical state. Exploring the anti-interference characteristics of the nervous system from the perspective its composition is a major direction in the study of neural networks.

Wang et al. (2011) and Wang et al. (2012) studied how the information transmission of the nervous system is affected by the phase synchronization between neurons and the neural coding in a variety of neural oscillator population networks. Wang (2010) studied the effect of neuronal synchronization on neural signal transmission. The results show that the higher the neuronal synchronization, the stronger the consistency of the transmitted signal. Liu et al. (2024) constructed a new memristive device, and the synaptic plasticity mechanism was successfully realized. The study found that the device applied to the neural network can improve network performance. This study can confirm the influence of the synaptic plasticity mechanism on the anti-interference of neural networks from the side. Guo et al. (2017) studied the significance of the biological mechanism of synaptic plasticity (STDP) in small-world spiking neural networks. Studies have found that STDP has a regulatory effect on the transmission of neural information in biological neural networks. Kleberg et al. (2014) found that the plasticity of excitatory synapses and inhibitory synapses can work together. The two synaptic plasticity regulate the path selection of signal transmission in the neural network through synergy and regulate the synchronization effect of the spiking neural network. Liu et al. (2020) constructed a scale-free peak neural network (sfSNN) with small-world characteristics and explored the robustness mechanism of the neural network. Studies have found that the adaptive regulation of synaptic plasticity is an internal factor for network robustness. The research of Guo et al. (2024) aims to explore the anti-interference ability of brain-like models with biological attributes. A spiking neural network (SWSNN) simulating biological attributes was constructed using the Izhikevich neuron model and the synaptic plasticity model with time delay. The research results prove that the dynamic regulatory effect of synaptic plasticity makes the anti-interference effect of SWSNN superior to that of the general spiking neural network (SNN). Liu et al. (2021) constructed two Hopfield (HNN) neural networks and studied the synchronization of neural networks by numerical simulation. The results show that the network can produce different synchronizations according to the different synaptic coupling strengths. Liu et al. (2021) constructed two spiking neural networks with different clustering coefficients to study the anti-interference ability of the network. The experimental results show that the spiking neural network with a high clustering coefficient has a better anti-interference effect. Abhirup (Abhirup and Samarjit, 2018) explored the influence of networks with different structures on phase synchronization. The synchrony of Hindmarsh-Rose (HR) neurons under random, regular, small-world, scale-free, and modular network topologies was analyzed. The research finds that the network with a mixed pattern of high clustering coefficient and neutrality degree promotes better synchronization.

Most of the above-mentioned related studies use simplified neuron models such as Integrate-and-Fire (IF) and Hindmarsh-Rose (HR). The disadvantage of these simplified models is that they cannot well reflect the biological properties of neurons. For example, the IF neuron model mimics the threshold behavior of membrane potential but neglects subcellular-level ionic interactions, which fails to capture the impact of ion channel stochasticity on action potential waveforms. At the same time, there are few neurons used in the construction of the network (for example, the network composed of 51 neurons was constructed in Reference 15, while only several neurons were studied in Reference 12), which cannot reflect the complexity of the network. The HH model, on the contrary, by explicitly modeling voltage-gated ion channel dynamics, enables the direct investigation of interactions between internal neuronal states and external perturbations.

This study integrates biological Hodgkin–Huxley (HH) neuronal dynamics and chemical synaptic mechanisms to construct a Newman–Watts (NW) small-world network by scaling the system to 100 HH-model neurons, a relatively larger network compared to the typical prior simulations. The framework preserves biophysical fidelity while capturing network-level complexity. This combination of molecular-level ion channel dynamics, synaptic interactions, and structured connectivity bridges a critical gap in modeling neural robustness, which enables the reveal of how synaptic plasticity and network structure synergistically enhance anti-interference capabilities, demonstrating superior noise resilience compared to conventional spiking neural networks. This framework bridges molecular-level dynamics with macroscale network behavior, offering novel insights into how biological neural systems maintain functional accuracy under environmental and intrinsic noise.

## 2 Method

### 2.1 HH neuron model

In the 1950s, the HH model was proposed by Alan Hodgkin and Andrew Huxley (Hodgkin, 1952; Hodgkin and Huxley, 1952a,b,c). The original HH model was based on the data of the nerve stimulation potential in squid and then became a model prototype of nerve cells in many different physiological structures. The HH model proposed the concept of 'ion channel' for the first time. The 'ion channel' is used to explain the membrane potential change and the unidirectional conduction process of the neuron cells, which makes the HH model simulate the real characteristics of biological neurons to a certain extent. The HH model is a mathematical model. The model describes the membrane potential activity of neuronal cells through four first-order differential equations:


(1) $$\{\begin{array}{l} \frac{dm}{dt}=a_{m}(1-m)-b_{m}m\\ \frac{dh}{dt}=a_{h}(1-h)-b_{h}h\\ \frac{dn}{dt}=a_{n}(1-n)-b_{n}n\\ C\frac{dV}{dt}=G_{Na}m^{3}h(E_{Na}-V)\\ +G_{K}n^{4}(E_{K}-V)+G_{L}(E_{L}-V)+I \end{array}$$

In the above formula, V is the membrane potential of neuronal cells, and the initial value is the resting potential, that is, −65 mV. G_Na_ is the maximum conductance of the sodium ion channel, with a value of 120 mS/cm^2^; G_K_ is the maximum conductance of the potassium ion channel, with a value of 360 mS /cm^2^; G_L_ is the leakage conductance, with a value of 0.3 mS/cm^2^. E_Na_, E_K_, and E_L_ are the reversal electromotive forces of the sodium ion channel, potassium ion channel, and leakage current, and their values are 50 mV, −77 mV, and −54.5 mV, respectively. I is the external stimulation current. C is the membrane capacitance of neurons, and its value is 1 mS/cm^2^. m is the parameter of the activation process of the sodium channel, h is the parameter of the inactivation process of the sodium channel, and n is the parameter of the activation process of the potassium channel. α and β are rate constants, and their changes are only related to the change of membrane potential and have nothing to do with time. The formulas of α_m_, β_m_, α_h_, β_h_, α_n_, and β_n_ are as follows (Bazsó et al., 2003):


(2) $$\{\alpha_{\text{m}}\text{=}\frac{\text{0}.\text{1}\left(\text{V+40}\right)}{\text{1 }-\exp\left(\;-\;\frac{\left(\text{V+40}\right)}{\text{10}}\right)}\;\beta_{\text{m}}\text{= 4}\exp\left(-\frac{\left(\text{V+65}\right)}{\text{18}}\right)$$

(3) $$\{\alpha_{\text{h}}\text{=0}.\text{07}\exp\left(-\;\frac{\left(\text{V+65}\right)}{\text{20}}\right)\;\beta_{\text{h}}\text{=}\frac{\text{1}}{\text{1 }-\;\exp\left(-\;\frac{\left(\text{V+35}\right)}{\text{10}}\right)\text{+1}}$$

(4) $$\{\;\alpha_{\text{n}}\text{=}\frac{\text{0}.\text{01}\left(\text{V+55}\right)}{\text{1}-\exp\left(-\;\frac{\left(\text{V+55}\right)}{\text{10}}\right)}\;\beta_{\text{h}}\text{=0}.\text{125}\exp\left(-\frac{\left(\text{V+65}\right)}{\text{80}}\right)$$

### 2.2 Neurosynaptic model

A single neuron cannot complete the complex functions of the nervous system of humans or other vertebrates. To realize the complex functions of the biological nervous system, neurons need to form a neural network. The key to connecting neurons into a network is synapses. Synapses are the basis of complex functions, including learning, movement, and memory in the biological nervous system. It can be said that synapses play an important role in the realization of brain function (Bacci and Huguenard, 2006). For example, Rossoni et al. (2005) used the effect of time delay on the coupling of HH neurons. Experiments show that time delay can cause synchronous oscillation of neurons under certain conditions. In Yilmaz et al. (2013), the phenomenon of stochastic resonance in neural networks was studied. The results show that the network structure and synaptic connection act together on the occurrence of resonance. Reference Yu et al. (2017) studied the effect of adding mixed synapses and time delays on network stochastic resonance in small-world networks. The results show that the use of mixed synapses and time-lag small-world network stochastic resonance has been significantly enhanced. A considerable number of studies have shown that synapses play an important role in the transmission and processing of information in neural networks. The understanding of synaptic mechanisms helps us to understand the mechanism of the nervous system in depth (Kuramoto and Battogtokh, 2002; Xia and Qi-Shao, 2005; Wang and Shi, 2010; Li and Cao, 2011; Ma et al., 2017). Neurosynapses are divided into electrical synapses and chemical synapses. Chemical synapses and electrical synapses exist in the human nervous system. However, compared with electrical synapses, chemical synapses are more complex in structure, more powerful in performance, and ubiquitous in the human nervous system. Reference L'Espérance and Labib (2013) proposed and described a stochastic process synaptic model. Experiments show that the model can complete the synaptic transmission process. Reference Veletic et al. (2015) discussed the possibility of applying communication theory to synaptic research and designing synaptic models using communication theory. In Alam and Hasan (2016), VLSI circuits were used to simulate the effect of chemical synaptic transmission. It was found that synaptic transmission is a key step in neural signal processing. Reference Wang et al. (2016) used ML neurons to study the synchronization conditions of neurons under chemical synaptic conditions. Reference Yaghini Bonabi et al. (2014) proposed a method for efficient implementation of biological neural networks based on the HH model on field programmable gate array (FPGA). In this study, we will use electrical synapses and two chemical synapse models to study the anti-interference characteristics of neural networks, and compare and analyze the differences in anti-interference ability of different synaptic coupling networks.

#### 2.2.1 Chemical synapse model

In 1992, Hansel and Sompolinsky (1992) proposed a simple model to describe chemical synapses. Its expression is:


(5) $${\text{I}}_{\text{syn}}{\text{=G}}_{\text{syn}}{\text{H(V}}_{\text{pre}}\text{(t}-\tau \text{) }-\;{\text{V}}_{\text{thresh}}\text{)}$$

Among them, I_syn_ is the postsynaptic current, and the unit is μA/cm^2^. G_syn_ is the synaptic coupling strength, and the unit is mS/cm^2^. H is the Heaviside function. V_pre_ is the membrane potential of presynaptic neurons in mV. V_thresh_ is the synaptic threshold in mV. τ is the time delay of the signal between neurons, and the unit is mS.

In 1997, Rabinovich et al. studied the problem of neurons connecting through inhibitory synapses in the central nervous system of animals and proposed a chemical synaptic model with time-lag based on the mechanism of synaptic delay and neuronal excitation and inhibition (Rabinovich et al., 1997). Its mathematical expression is:


(6) $${\text{I}}_{\text{syn}}\text{= }{\text{G}}_{\text{syn}}\left(\text{V}-{\text{V}}_{\text{syn}}\right)\text{H}\left({\text{V}}_{\text{pre}}\left(\text{t}-\tau \right)\;-{\text{V}}_{\text{thresh}}\right)$$

In the formula, I_syn_ is the postsynaptic current, and the unit is μA/cm^2^. G_syn_ is the synaptic coupling strength, and the unit is mS/cm^2^. E_syn_ is a synaptic reversible potential in mV. H is a Heaviside function, which represents the instantaneous change of current or voltage in the circuit. The V_thresh_ is the synaptic threshold associated with the Heaviside function. τ is the time delay of synaptic transmission signal. From the perspective of the formula, the Rabinovich chemical synaptic model has more biological properties based on the synaptic delay and the excitatory inhibition mechanism of neurons. Compared with the Hansel chemical synapse model, the Rabinovich chemical synapse model provides a postsynaptic neuron feedback mechanism. That is, the real synaptic current intensity is not only determined by the presynaptic current (*V*_*pre*_(*t*−τ)−*V*_*thresh*_) but also by the difference between the postsynaptic membrane potential and the synaptic reversible potential (*V*−*E*_*syn*_). Adding this difference to the synaptic equation can suppress the abnormal fluctuation of postsynaptic potential caused by noise and can enhance the weak signal while suppressing the strong noise. Therefore, the anti-interference effect of the more complex Rabinovich chemical synaptic model should be better.

**Figure 1:**
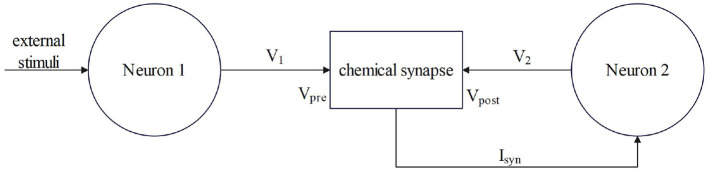
The process by which chemical synapses couple two neurons.

Synaptic coupling connects two or more neurons together to complete the conduction of action, resting potential, and stimulation between neurons. The input of chemical synaptic coupling is the state of a presynaptic neuron, and the output is the stimulation signal to a postsynaptic neuron, that is synaptic current. The process by which the chemical synapses couple two neurons is shown in Figure 1.

Neuron 1 is a presynaptic neuron. After external stimulation, the potential inside and outside the membrane changes to produce a membrane potential V_1_, which is the input of the synapse, that is, the state of the presynaptic neuron. After the conduction of the membrane potential V_1_ through chemical synapses, synapses produce synaptic current (I_syn_), which is transmitted to postsynaptic neurons as the input to neuron 2. The postsynaptic neuron-neuron 2 will feed back to the synapse after the membrane potential (V_2_) is generated and the transmission of the stimulation signal between the neurons is completed. Studies have shown that chemical synaptic coupling is unidirectional coupling, that is, its conduction is asymmetric, and the structure is also asymmetric. Therefore, two neurons coupled by a chemical synapse can only transmit stimulus signals in the direction of postsynaptic neurons.

#### 2.2.2 Electrical synapse model

Compared with chemical synapses, the electrical synapse model is simpler, and the mathematical description of the model is as follows (Wang et al., 2007):


(7) $${\text{I}}_{\text{ele}}{\text{=g}}_{\text{ele}}\left({\text{V}}_{\text{pre}}\;-\;{\text{V}}_{\text{post}}\right)$$

**Figure 2:**
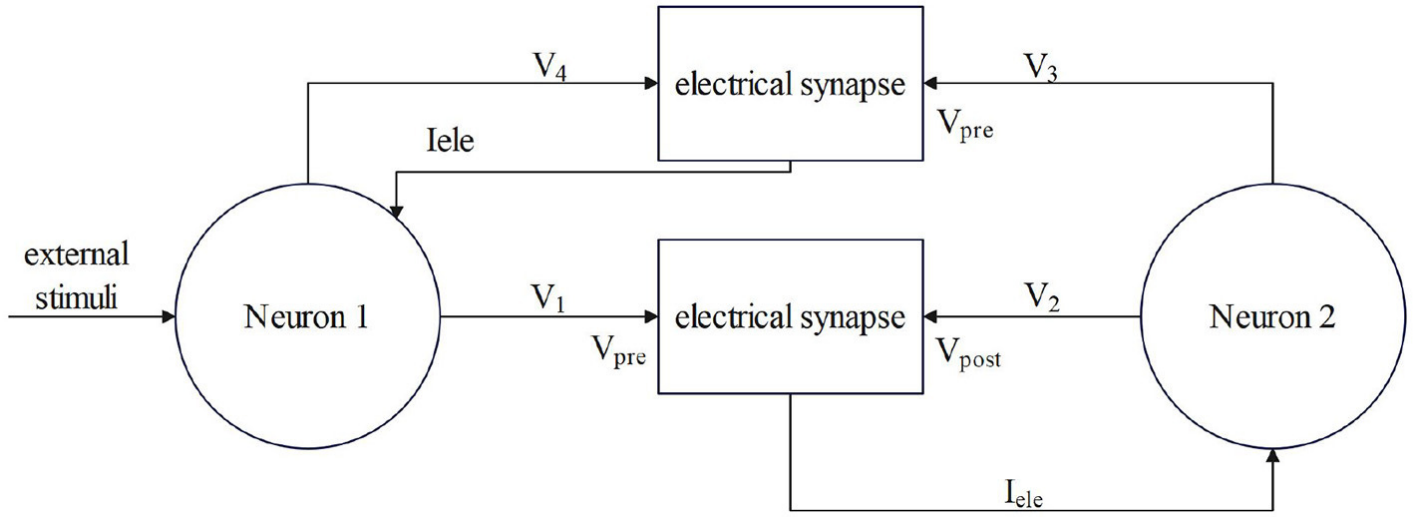
The process by which electrical synapses couple two neurons.

The g_ele_ is the coupling strength in mS/cm^2^. V_pre_ and V_post_ represent presynaptic and postsynaptic neuronal membrane potentials, respectively, i mS/cm^2^. The electrical contact is bidirectional coupling, and the signal can be transmitted in both directions. The model of coupling two neurons is shown in Figure 2.

### 2.3 Neural network model

Because of the asymmetric structure and conduction of chemical synapses, the neural network model can be decomposed into a simple model of one-way transmission of stimulus signals by multiple double neurons. The mathematical expression of the coupling of two HH neurons through the chemical synapse model with time delay is as follows:


(8) $$\begin{array}{l} \text{C}({\text{dV}}_{1}){\text{/dt=G}}_{\text{Na}}{\text{m}}_{1}^{\text{3}}{\text{h}}_{1}({\text{E}}_{\text{Na}}\;-\;{\text{V}}_{1}){\text{+G}}_{\text{K}}{\text{n}}_{1}^{\text{4}}({\text{E}}_{\text{K}}-{\text{V}}_{1})\text{+}\\ \;{\text{G}}_{\text{L}}({\text{E}}_{\text{L}}{\text{V}}_{1}){\text{+I}}_{\text{ext}} \end{array}$$

(9) $$\begin{array}{l} \text{C}({\text{dV}}_{\text{2}}){\text{/dt=G}}_{\text{Na}}{\text{m}}_{\text{2}}^{\text{3}}{\text{h}}_{\text{2}}({\text{E}}_{\text{Na}}\;-\;{\text{V}}_{\text{2}}){\text{+G}}_{\text{K}}{\text{n}}_{\text{2}}^{\text{4}}({\text{E}}_{\text{K}}-{\text{V}}_{\text{2}})\text{+}\\ \;{\text{G}}_{\text{L}}({\text{E}}_{\text{L}}{\text{V}}_{\text{2}}){\text{+I}}_{\text{syn}} \end{array}$$

(10) $${\text{I}}_{\text{syn}}{\text{=G}}_{\text{syn}}\left(\text{V}-{\text{V}}_{\text{syn}}\right)\text{H}\left({\text{V}}_{\text{pre}}\left(\text{t}-\tau \right)\;-\;{\text{V}}_{\text{thresh}}\right)$$

The main difference between this group of formulas and the membrane potential formula of the HH neuron model formula above is that the stimulation current is applied to the presynaptic neuron, and the postsynaptic current is increased to the postsynaptic neuron—Formula (10).

The I_ext_ in (8) is the stimulation current applied to the presynaptic neuron. In the neuronal network, only the first neuron is externally stimulated, and the remaining neurons are stimulated by the stimulation signal generated by the first neuron. In Equation 9, I_syn_ is added after the basic equation of the membrane potential of HH neurons. I_syn_ is the postsynaptic current. According to the previous value of I_syn_, it will be affected by the membrane potential of postsynaptic neurons, and the direction of the current is the direction of the next neuron.

The above formulas (8), (9), and (10) are the mathematical models of two HH neurons before and after chemical synaptic coupling, and the ion channel parameters such as m, h, and n in the formula are consistent with the previous description of a single HH neuron model. In this study, the initial value of V is−65mV, and the initial values of m, h, and n are 0.05293, 0.5961, and 0.3177, respectively. These three initial values can be calculated by the rate function of m, h, and n. The specific values of other parameters in the model are shown in Table 1.

**Table 1:** Numerical simulation model parameters of the neural network.

**Variable name**	**Symbol**	**Value unit**
Membrane capacitance	C/Cm^2^	1 μF/cm^2^
The maximum conductance of sodium ion channel	G_Na_	120 mS/cm^2^
The maximum conductance of potassium ion channel	G_K_	360 mS/cm^2^
leakage conductance	G_L_	0.3 mS/cm^2^
Sodium ion channel reversal electromotive force	E_Na_	50 mV
Potassium ion channel reversal electromotive force	E_K_	−77 mV
Leakage current reversal electromotive force	E_L_	−54.5 mV
Reversible synaptic potential	E_syn_	−70 mV–−52 mV
Postsynaptic membrane potential	V_syn_	0 mV
synaptic threshold		−52 mV

The initial conditions of the model simulation are shown in Table 2.

**Table 2:** The initial conditions of model simulation.

**Condition**	**Values and units**
Simulation time of two synaptic coupled neurons	100 mS
Square wave amplitude	10 μA/cm^2^
Square wave frequency	0.05 Hz
100 neural network simulation time	300 mS
Sine wave amplitude	60 μA/cm^2^
sinusoidal undulation frequency	50 Hz
Sine wave offset	30 mS
Gaussian white noise mean	0
Gaussian white noise standard deviation	20

When the electrical synapse is coupled to the neuron, the stimulation signal can be transmitted in two directions, as reflected in the formulas for coupling two HH models, similar to Formulas (8), (9), and (10): the current after the electrical synapse is added after Formula (8).

**Figure 3:**
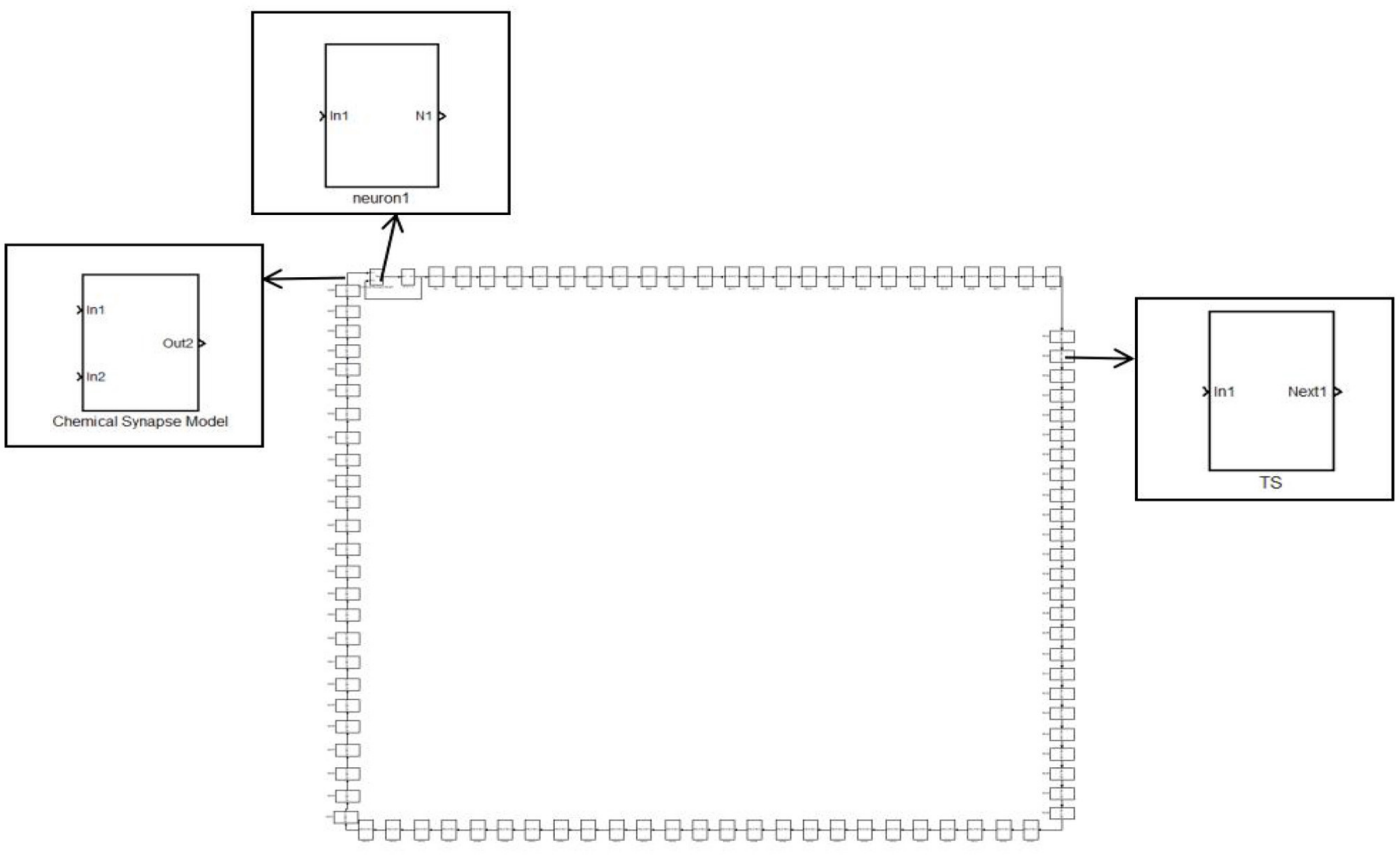
A simple ring network structure composed of 100 neurons.

A single neuron cannot complete the complex functions of the nervous system. To achieve complex functions, it must rely on a neural network. The last neuron is connected to the first neuron through synapses, and other neurons are connected in turn through synapses. Each neuron only receives the stimulation of the previous neuron, which constitutes a ring network. The synaptic coupled ring network model is shown in Figure 3. It consists of three parts: the HH neuron module, the Chemical Synapse Model module, and the TS module (encapsulating neurons and synapses).

The brain is a complex system composed of multiple functional regions, and it features a large number of complex network topologies in this complex system. The ring network topology is not enough to describe the complexity of the neural system structure, so this study continues to study the effects of synapses and structures on the discharge of complex topological networks on the basis of the ring network.

**Figure 4:**
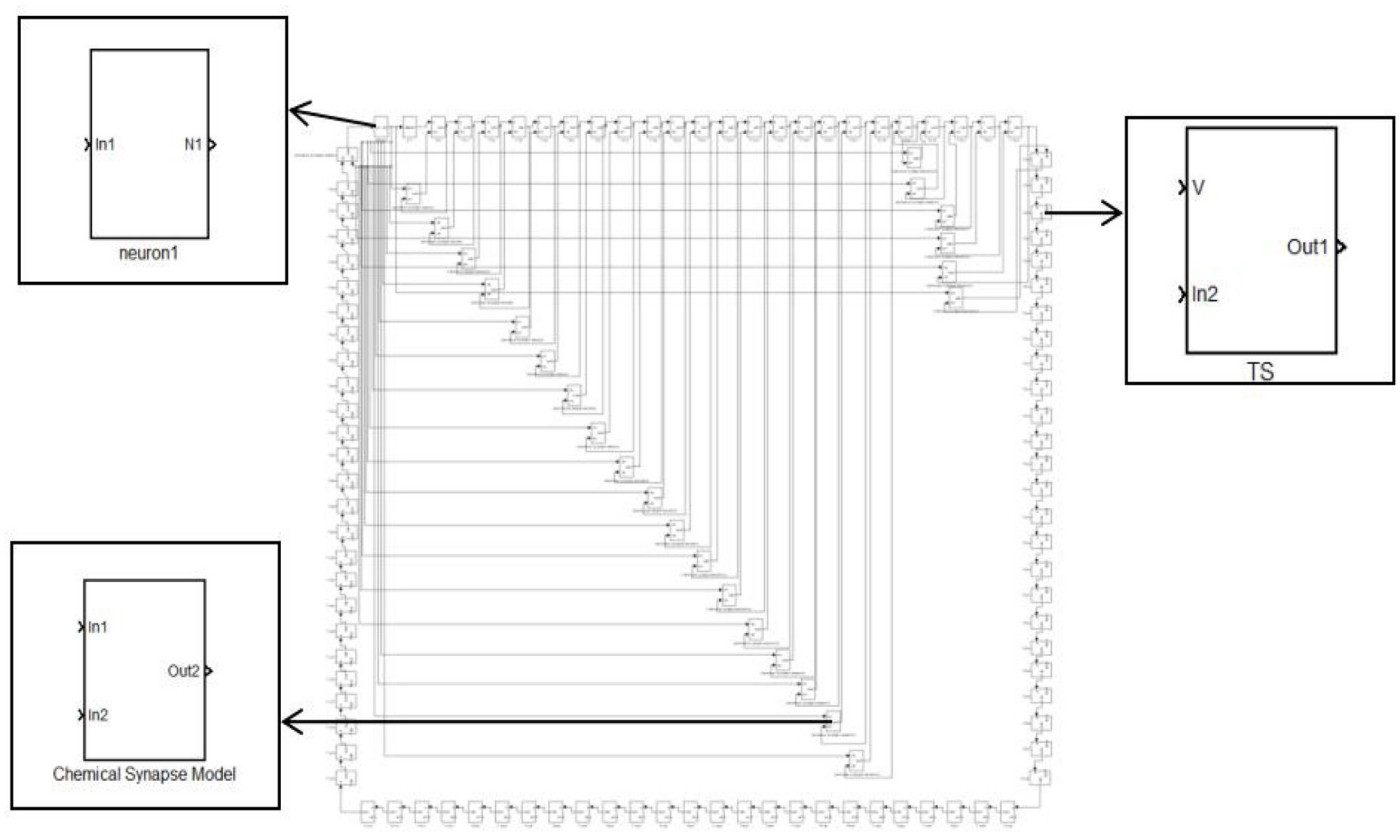
Topology of the 100 neurons network.

The small-world network has a shorter average path length and a higher clustering coefficient network. The NW small-world network increases the link between any two nodes of the ring network with the probability of p. When P=0, it is a ring network, and when p=1, it is a global coupling network. The first neuron receiving external stimulation is used as the central node, and it is connected with 24 other neurons that are not directly connected by synapses to form a complex network topology. The NW small-world network is constructed, and its topology is shown in Figure 4. In Simulink modeling, the same specific encapsulation module as the ring network topology is used to build the topology. The difference is that more synaptic modules are used to build the topology.

This study employs Simulink, the block diagram environment within MATLAB mathematical software, for modeling and simulation. Simulink is a visual numerical simulation calculation tool of MATLAB. The MATLAB code and the model can be combined to realize the numerical simulation of the dynamic system. The model library is divided into a standard library and a professional library. The standard library is the necessary model library in modeling and simulation, and the professional library is developed on the standard model library to meet the needs of a certain field. This study uses a standard model library to build models.

The HH neuron model and synaptic model were built and packaged. The neuron model and synaptic model are encapsulated into a modular single system, which is not only convenient for the construction of neural networks and the replacement and change of different synaptic structures and parameters but also convenient for intuitive response to the relationship between neurons, synapses, and other unit modules in the model.

**Figure 5:**
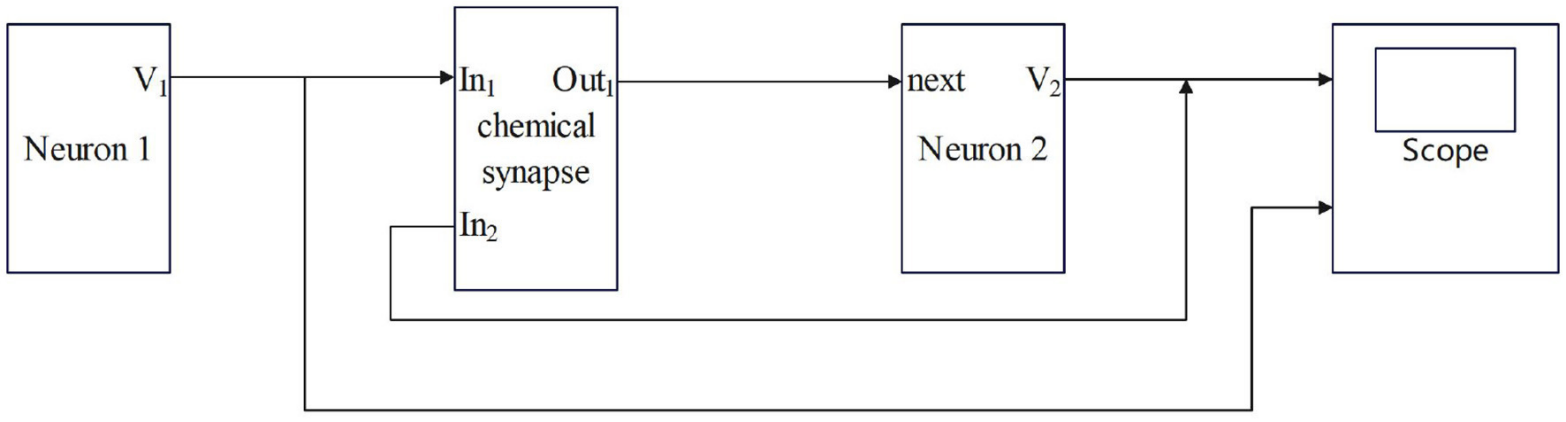
Simulink Modeling of Chemical Synapses Coupling Two Neurons. (next: Neuronal signal output; In_1_: Front-end signal receiving signal port; In_2_: Feedback signal receiving signal port; Out_1_: Postsynaptic current output port; scope: Display neuron action potential waveform).

**Figure 6:**
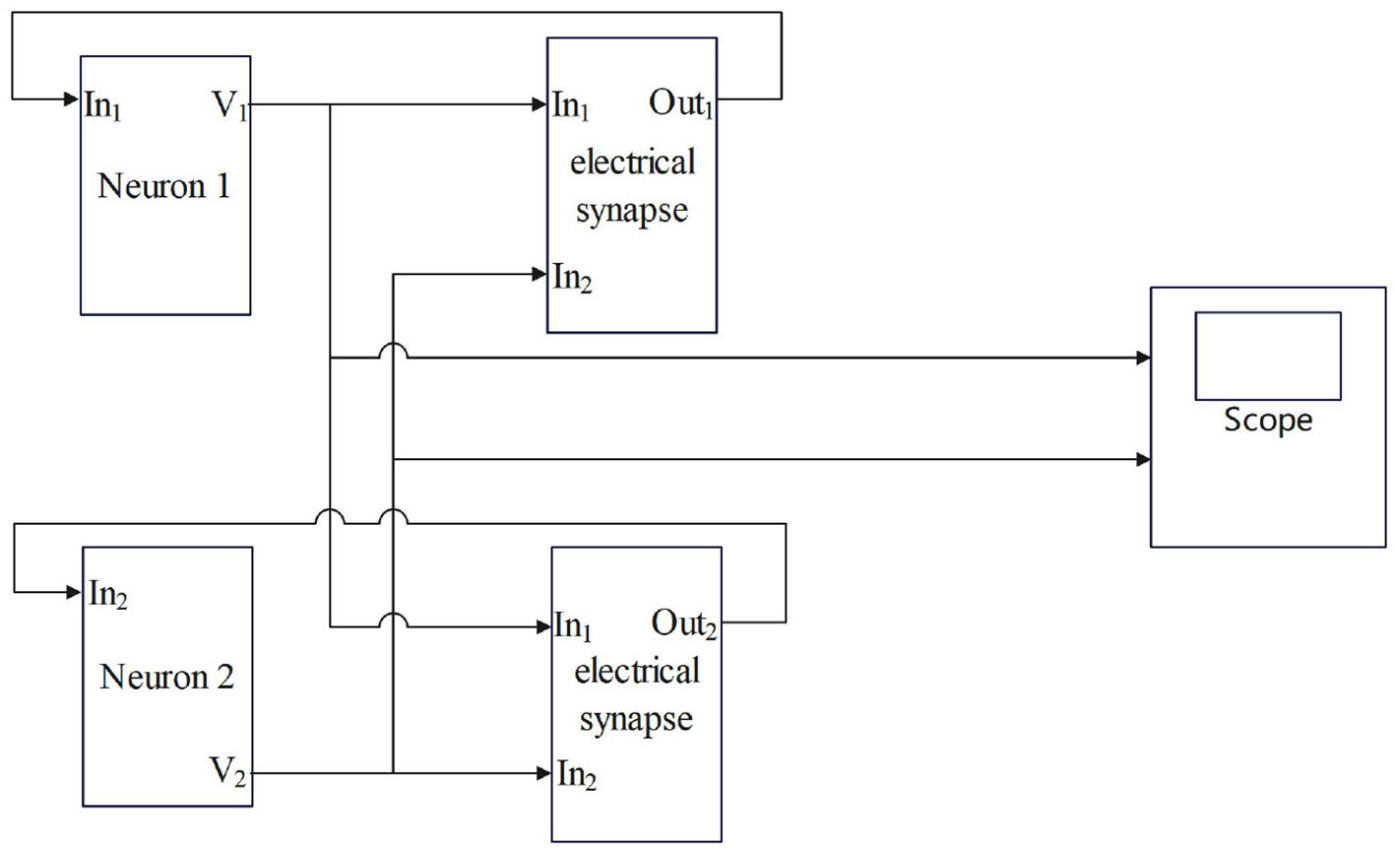
Simulink modeling of electrical synapse coupling two neurons. (Neuron-In_1_: Neuron receiving signal port; In_1_: Electrosynaptic receiving neuron1 signal port; In_2_: Electrosynaptic receiving neuron2 signal port; Neuron-V_1_,V_2_: Neuron output signal port; Out_1_: Electrical contact output signal port; scope: Display neuron action potential waveform).

In Figures 1, 2, the process of chemical and electrical synapses coupling two neurons is constructed to verify whether the action and resting potential of the model are consistent with the potential conduction process of real neurons. The Simulink modeling of synaptic coupling of two neurons in Figures 1, 2 is shown in Figures 5, 6. The 'stimulation' is the external current signal, and neuron 1 is the neuron that receives the external stimulation and transmits the stimulation signal through the synapse. Neuron 2 only received synaptic transmission of stimulus information without additional stimulation.

## 3 Simulation results

### 3.1 Action potential simulation in networks using synaptic connection between two neurons

HH neurons show a variety of characteristics when receiving external stimulation signals, and the most prominent feature is the characteristics of neuronal action potential firing. The action potential is the electrical activity generated by neurons when they are stimulated and transmit signals. It is an instantaneous change in the internal and external potentials of neurons, usually manifested as rapid voltage changes. Action potential has three most important characteristics: 'all or none', non-superposition, and non-attenuation conductivity.

**Figure 7:**
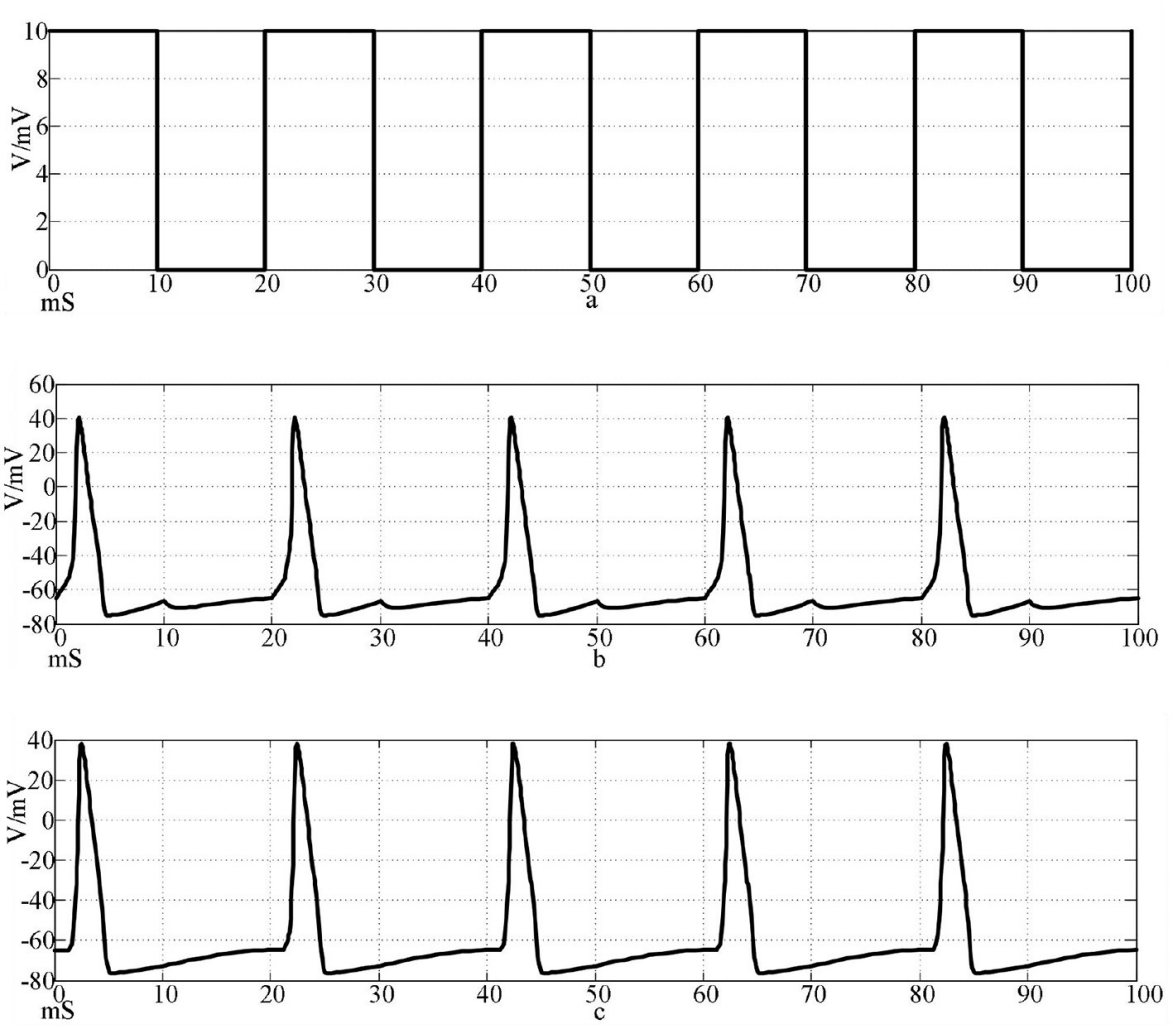
Action potentials of two neurons coupled by chemical synapses stimulated by a square wave signal. [**(a)** Square wave stimulation signal; **(b)** neuron 1 action potential waveform; c: Neuron 2 action potential waveform].

A square wave with an amplitude of 10 μA/cm^2^ and a frequency of 0.05 Hz is used as the input signal, and the formula is $I_{ext}(t)=\{\begin{array}{c} 10\left(20k<t<20k+10\right)\\ 0\left(20k+10<t<20(k+1)\right) \end{array}\;$, the simulation time is 100 mS. The oscilloscope module is added to the model in Simulink to display the simulation results of the two neurons. The numerical simulation results of the action potential of two neurons connected by a chemical synapse are shown in Figure 7.

**Figure 8:**
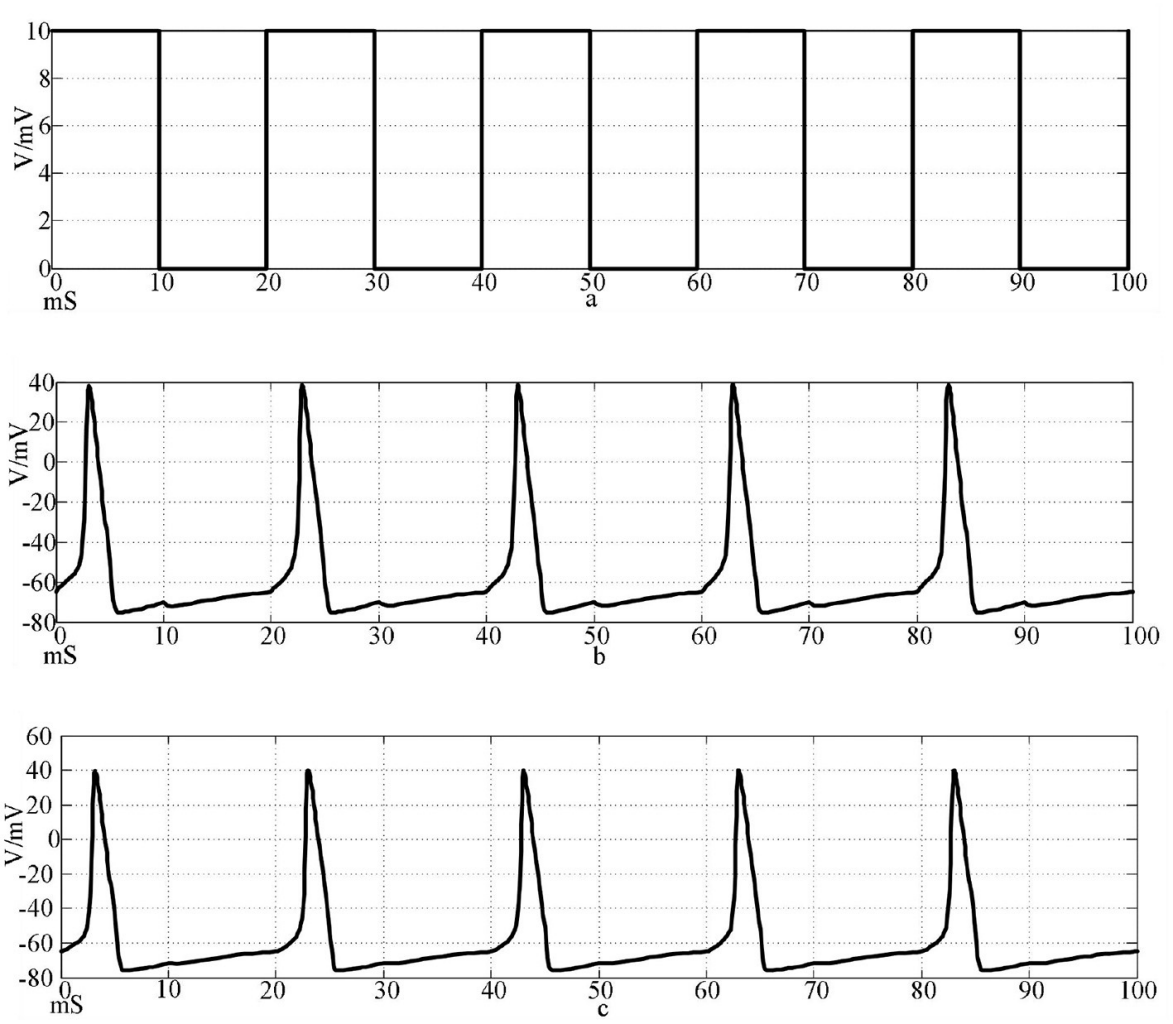
Action potentials of two neurons coupled to electrical synapses stimulated by a square wave signal. [**(a)** Square wave stimulation signal; **(b)** neuron 1 action potential waveform; **(c)** Neuron 2 action potential waveform].

Under the same stimulation signal, the numerical simulation results of the action potential of two neurons connected by two electrical synapses are shown in Figure 8.

The top waveform in Figures 7, 8 is the waveform of square wave stimulation information, the middle waveform is the action potential waveform generated by the neurons directly receiving the stimulation information, and the bottom waveform is the waveform generated by the postsynaptic neurons. It can be seen that the two neurons connected by the two synapses produced complete action potentials.

### 3.2 Stimulation signal and interference signal

**Figure 9:**
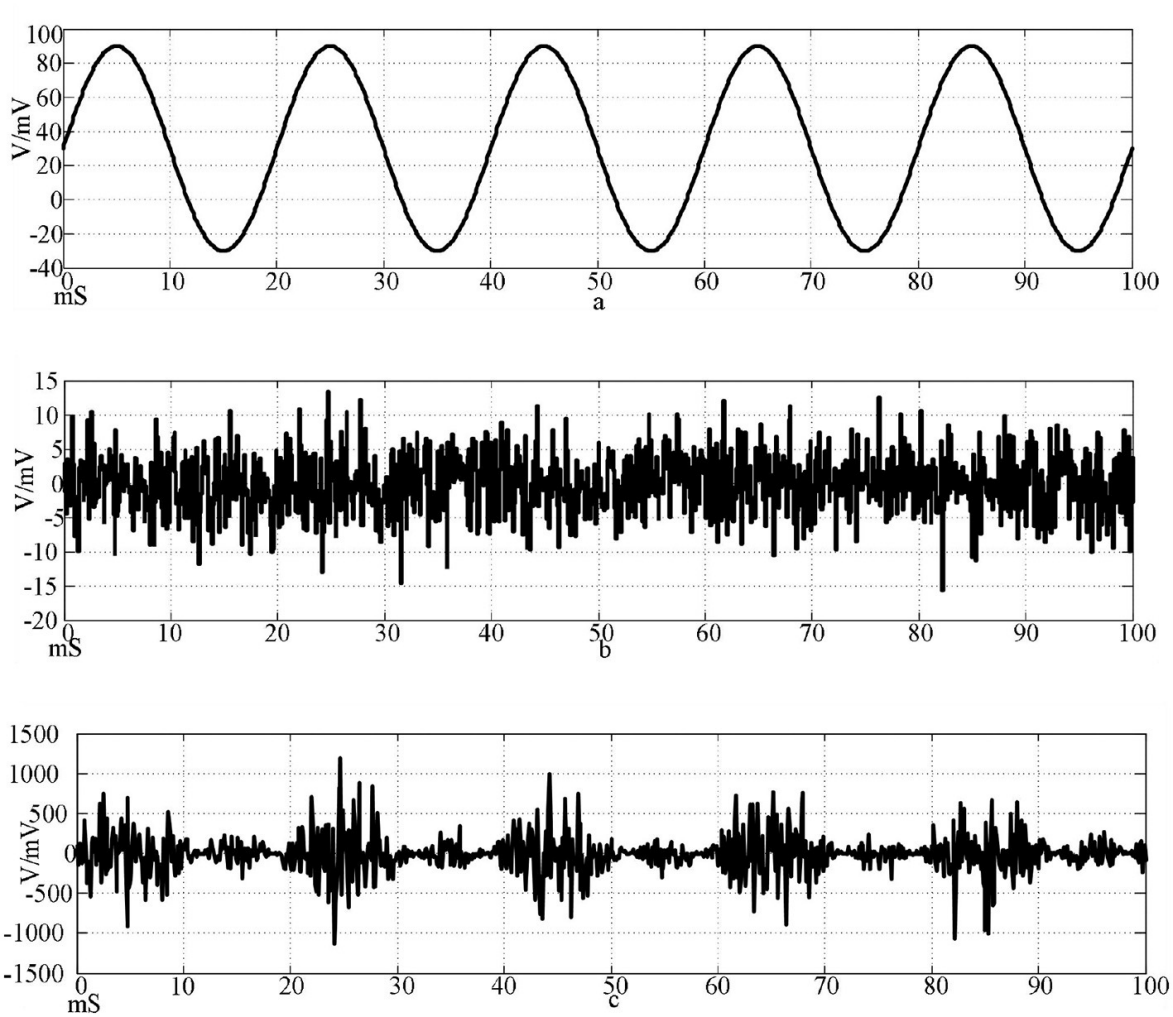
Input signal waveform. [**(a)** Sine wave stimulation signal; **(b)** Gaussian white noise interference signal; **(c)** Stimulus signal after superimposed interference].

The simulation results of the synaptic connection of two neurons show that the method is feasible and can be used for subsequent research. The sine wave I_ext_=60 sin(0.1πt)+30 with amplitude of 60μA/cm^2^, frequency of 50 Hz, and offset of 30 mS was selected as the stimulus signal input. A Gaussian white noise with a mean value of 0 and a standard deviation of 25 is selected as the interference signal. Superimpose the input with the stimulation signal to explore the anti-interference ability of the network. The stimulation signal, the interference signal, and the stimulation signal after the superposition of the interference are shown in Figure 9.

The top of Figure 9 is the waveform of sine wave stimulation signal I_ext_=60sin(0.1πt)+30; the waveform of Gaussian white noise interference signal with an intermediate mean of 0 and a standard deviation of 20; the bottom is the stimulation signal superimposed with the interference signal.

### 3.3 Firing patterns in networks using hansel's chemically synaptic coupling model

Studies have shown that neurons are not fighting alone but rather through the synchronous discharge of neuronal clusters, that is, neural oscillations to achieve specific functions. Synchronized firing of neurons is an important feature of real biological neuronal networks, which is closely related to the functional realization of the nervous system. In the nervous system of vertebrates, especially humans, synchronous discharge is an important factor in the function of advanced functions such as memory and emotion. At the same time, the synchronous discharge of the neuronal network is also the key to the operation of anti-interference function of the biological nervous system.

**Figure 10:**
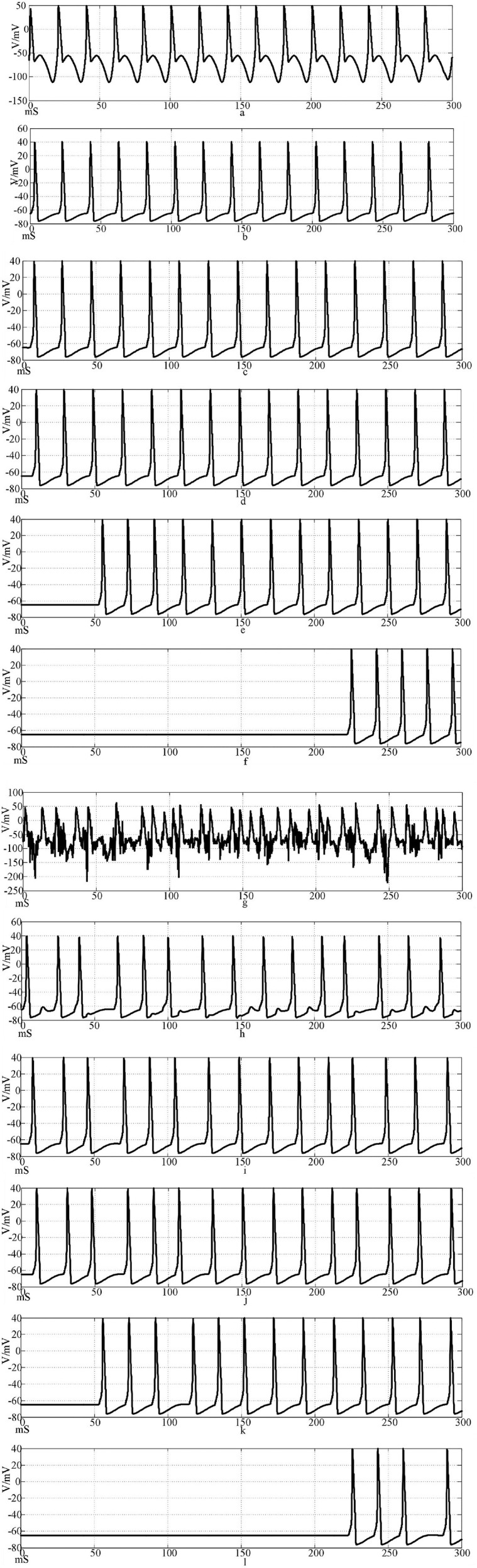
Waveforms of neuronal potentials in a ring network coupled by the Hansel synaptic model.[**(a–f)** Action potential waveforms of 1, 2, 4, 5, 25, and 100 neurons without interference were recorded; **(g–l)** The action potential waveforms of neurons 1, 2, 4, 5, 25, and 100 were interfered].

Using Hansel chemical synapses and 100 neurons, a network of 100 neurons is constructed in Simulink according to the topology of Figures 3, 4. The input stimulus signal is selected as the sine wave in Figure 9 and the sine wave superimposed with Gaussian white noise interference. The simulation time is set to 300 mS, the synaptic coupling strength is set to 0.1 mS/cm^2^, and the synaptic threshold is set to−52 mV. The first neuron in the network is set to receive the stimulation signal applied by the outside world or the stimulation signal after superimposed interference. Figures 10a–f is the discharge waveform of neurons 1, 2, 4, 5, 25, and 100 in the ring network after the input of the sine wave stimulation signal. It can be seen that the neurons in the ring network generate stable action potentials. It shows the action potential firing process and 'all or no' characteristics of depolarization, antipolarization, repolarization, and hyperpolarization. Figures 10g–l is the discharge waveform of neurons 1, 2, 4, 5, 25, and 100 after the sinusoidal input signal superimposed with Gaussian white noise interference input ring network. It can be seen that the firing of the first neuron action potential in the ring network after the sinusoidal input signal superimposed with interference reflects obvious interference. The specific performance is that an obvious subthreshold reaction is produced, and the hyperpolarization process is strengthened. The delay of subsequent neuron potential release in the network is lower than that of synchronous discharge, but the anti-interference effect is more obvious. It can be seen from Figures 10h, i that the second neuron is only slightly disturbed after synaptic transmission, and the fourth neuron is completely undisturbed. This result shows that the interference signal can be effectively reduced after synaptic transmission through the Hansel synaptic coupling network.

**Figure 11:**
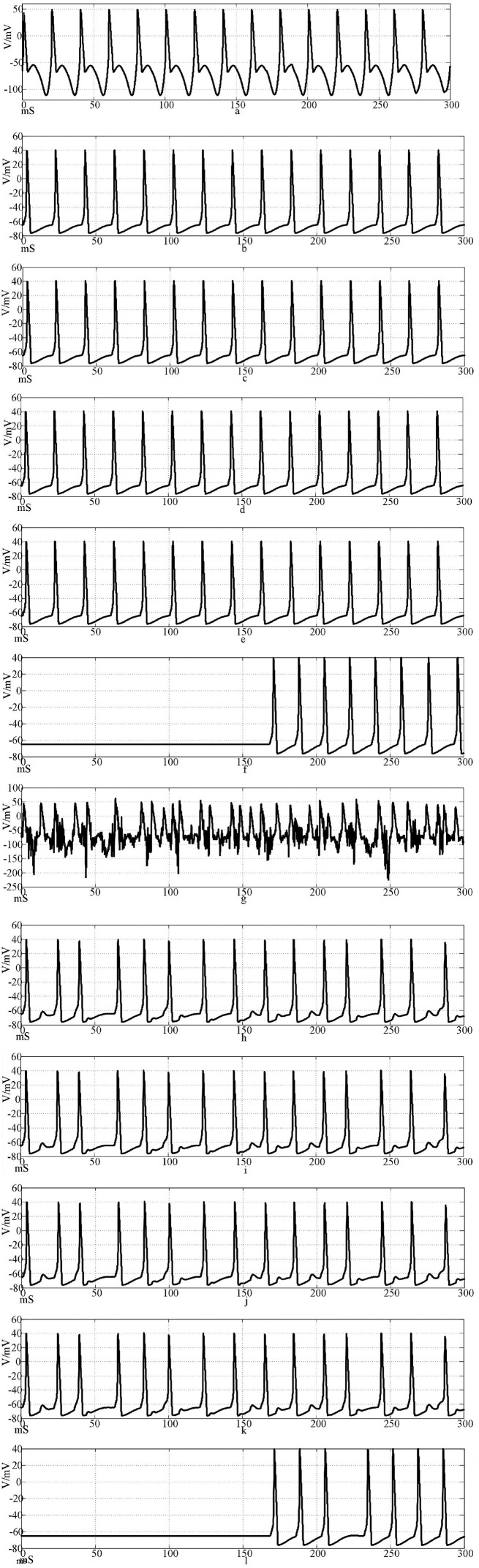
The waveform of neuronal potential in NW small-world network coupled by the Hansel synaptic model. [**(a–f)** Action potential waveforms of 1, 2, 4, 5, 25, and 100 neurons without interference were recorded; **(g–l)** The action potential waveforms of neurons 1, 2, 4, 5, 25, and 100 were interfered]..

Figure 11 shows the action potential waveforms of neurons 1, 2, 4, 5, 25, and 100 in the network after the network topology becomes a more complex NW small-world network. Figures 11a–f is the input of the sine wave stimulation signal. Comparing the waveforms of Figures 10a–f, 11a–f, it can be seen that when the network topology becomes an NW small-world, the delay of subsequent neuron potential firing in the network is significantly reduced, and the degree of synchronous discharge of the network is significantly increased. Figures 11g–l, is the stimulus signal input of sine wave superimposed with Gaussian white noise interference. Comparing the waveforms of Figures 10g–l, 11g–l, it can be seen that the interference degree of the first neuron action potential in the network under the same external interference is almost the same, but the waveforms of the subsequent neurons are slightly different. First, the delay of subsequent neuron potential release is reduced, and second, the synchronization degree of potential release is improved. From Figures 11h–k, it can be seen that the second neuron in the network has a slight subthreshold response, and the fourth, fifth, and 25th neurons all produce the same subthreshold response synchronously. This phenomenon does not appear in the waveform of Figures 10h–k. This result shows that using complex topology to increase the links between neurons that are not directly connected in the network can improve the synchronization degree of the neural network.

Under the condition that the above conditions remain unchanged, to further quantify the experimental results, the effects of different topologies and synapses on the synchronization and anti-interference effects of neuronal networks are compared. The Pearson correlation coefficient method was used to calculate the correlation between Neurons 1 and 25, neurons 5 and 45, neurons 15 and 30, and neurons 20 and 35 in ring network and NW small-world network, and the degree of network synchronization was quantitatively analyzed. The correlation coefficient is an index to measure the linear correlation between two random variables, which can make the data intuitive and easy to understand. Its mathematical formula is as follows (Liu et al., 2021; Maulana et al., 2023; Xu and Deng, 2018):


(11) $$\begin{array}{c} \text{r}=\frac{\overset{\text{n}}{\underset{\text{i}=1}{\sum }}\left({\text{x}}_{\text{i}}-\bar{\text{x}}\right)\left({\text{y}}_{\text{i}}-\bar{\text{y}}\right)}{\sqrt{\overset{\text{n}}{\underset{\text{i}=1}{\sum }}{\left({\text{x}}_{\text{i}}-\bar{\text{x}}\right)}^{2}•\overset{\text{n}}{\underset{\text{i}=1}{\sum }}{\left({\text{y}}_{\text{i}}-\bar{\text{y}}\right)}^{2}}} \end{array}$$

In the formula, r is the degree of correlation between the firing of two neuron action potentials. The closer the absolute value of r is to 1, the greater the correlation is. On the contrary, the closer to 0, the smaller the correlation is. *x*_*i*_ and *y*_*i*_ represent the ith data corresponding to the two groups of samples that calculate the correlation coefficient, and represent the mean value of each group of samples, and n is the amount of data of the sample.

The simulation time is set to 300 mS, and the correlation coefficient of action potential numerical calculation is collected. The calculation results are shown in Table 3.

**Table 3:** Correlation coefficient of Hansel's chemical synaptic coupling network.

**Condition**	***r*(1–25)**	***r*(15–30)**	***r*(20–35)**	***r*(5–45)**
Ring network topology, sine wave stimulation	0.236	0.471	0.191	0.168
Ring network topology, sine wave superposition interference	0.086	0.067	0.086	0.01
NW small-world network topology, sine wave	0.563	0.539	0.248	0.243
NW small-world network topology, sine wave Superimposed interference	0.163	0.096	0.090	0.086

The calculation results show that the synchronization of neuron action potential in the network is greatly reduced after the sinusoidal stimulation signal is superimposed with Gaussian white noise interference, which indicates that the anti-interference effect of Hansel chemical synaptic coupling network with a simple mathematical formula is not good.

### 3.4 Firing patterns in networks using rabinovich's chemically synaptic coupling model

The synapses of the coupled neuronal network were changed from Hansel chemical synapses to Rabinovich chemical synapses. According to the topological structure of Figures 3, 4, a network of 100 neurons was constructed in Simulink. The input stimulus signal is selected as the sine wave in Figure 9, and the sine wave is superimposed with Gaussian white noise interference. The simulation time is set to 300 mS, the synaptic coupling strength is set to 0.1 mS/cm^2^, and the synaptic threshold is set to−52 mV. The first neuron in the network is set to receive the stimulation signal applied by the outside world or the stimulation signal after superimposed interference. The effects of Rabinovich chemical synapses on network synchronous discharge and anti-interference ability were studied.

**Figure 12:**
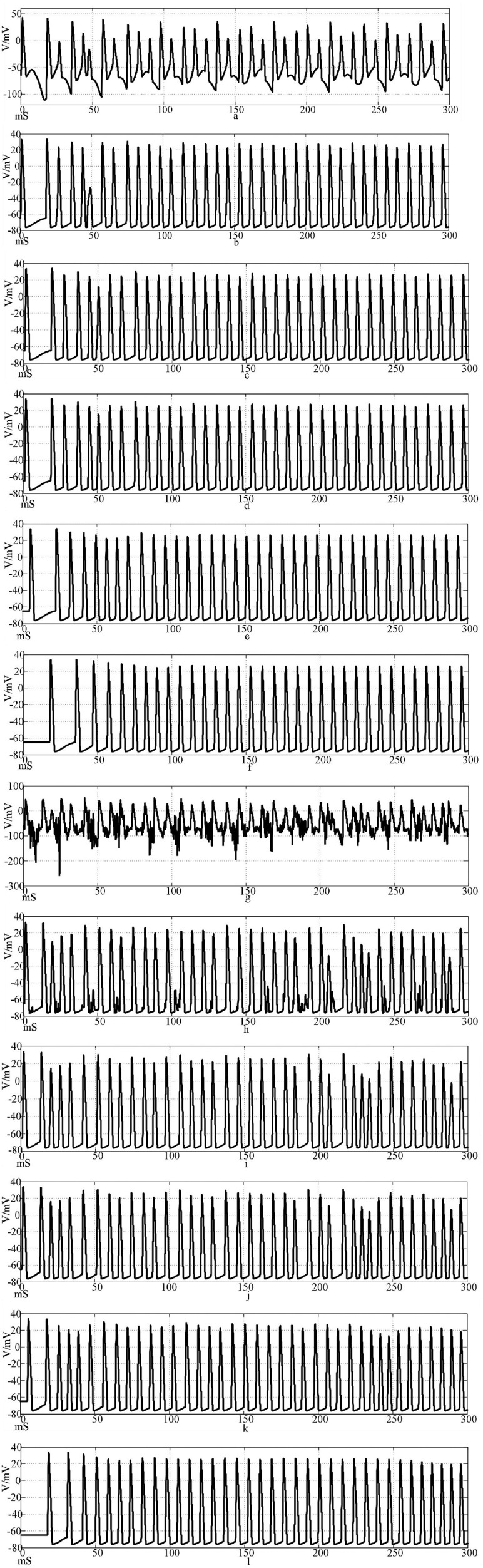
Waveforms of neuronal potentials in a ring network coupled by the Rabinovich synaptic model. [**(a–f)** Action potential waveforms of 1, 2, 4, 5, 25, and 100 neurons without interference were recorded; **(g–l)** The action potential waveforms of neurons 1, 2, 4, 5, 25, and 100 were interfered].

Figures 12a–f is the firing waveforms of neurons 1, 2, 4, 5, 25, and 100 in the Rabinovich synaptic coupling ring network after the input of the sinusoidal stimulation signal and the neurons in the network generate stable action potentials. The action potential firing process of depolarization, reverse polarization, repolarization, and hyperpolarization is complete, reflecting the 'all or no' characteristics. Figures 12g–l is the discharge waveforms of neurons 1, 2, 4, 5, 25, and 100 after the input of the Rabinovich synaptic coupling ring network by the sine wave superposition of Gaussian white noise interference. The firing of the first neuron potential in the network after the stimulation signal superposition interference reflects obvious interference and produces an obvious subthreshold response. There is a delay in the subsequent neuronal potential firing in the network, and the degree of synchronous discharge is low. However, the anti-interference effect is obvious. From Figures 12b, c, it can be seen that after synaptic conduction, two neurons are greatly reduced by interference, and only a small amount of subthreshold reaction is produced, while the fourth neuron is completely unaffected. This result shows that the interference signal can be effectively reduced after synaptic transmission through the Rabinovich synaptic coupling network. By comparing Figures 10, 12, it can be found that the Rabinovich synaptic coupling network has a lower potential firing delay than the Hansel synaptic coupling network except for the first neuron.

**Figure 13:**
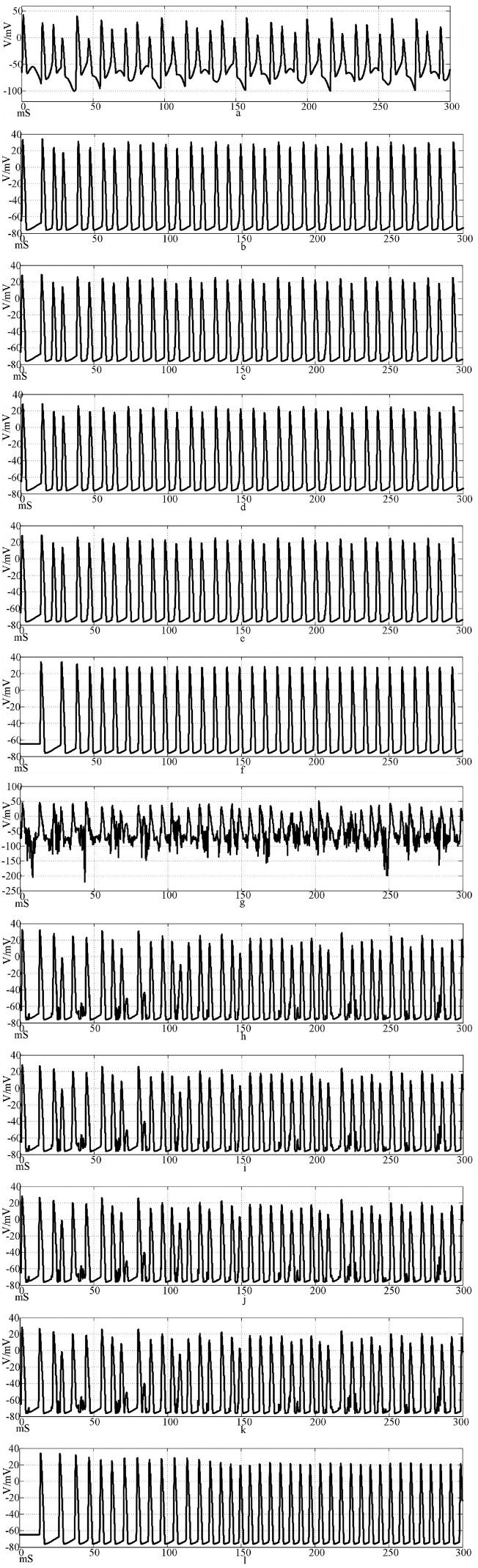
The waveform of neuronal potential in NW small-world network coupled by Rabinovichl synaptic model. [**(a–f)** Action potential waveforms of 1, 2, 4, 5, 25, and 100 neurons without interference were recorded; **(g–l)** The action potential waveforms of neurons 1, 2, 4, 5, 25 and 100 were interfered].

Figure 13 shows the action potential waveforms of neurons 1, 2, 4, 5, 25, and 100 in the network after the network topology becomes a more complex NW small-world network. Figures 13a–f is the input of the sine wave stimulation signal. Comparing the waveforms of Figures 12a–f, 13a–f, it is found that when the network topology becomes an NW small-world, in addition to the decrease of the delay of the subsequent neuron potential release and the increase of the degree of synchronous discharge, the neuron action potential in the network is more regular. Figures 13g–l is the stimulation signal input of sine wave superimposed with Gaussian white noise interference. Compared with Figures 12g–l, 13g–l, the degree of interference to the action potential of the first neuron in the network under the superimposed interference is almost the same, but the waveforms of subsequent neurons are different. First, the delay of subsequent neuron potential release is reduced, and second, the synchronization degree of action potential release is improved. It can be seen from Figures 13h–k that there is a subliminal response in neuron 2 in the small-world network. Similar to the Hansel synaptic coupled small-world network in Section 2.3, the 4th, 5th, and 25th neurons all produce the same subliminal response as neuron 2. This phenomenon does not appear in Figures 12h–k waveform. This result corroborates the experimental results of the small-world network in Section 2.3. Using complex topologies to increase the links between non-directly connected neurons in the network can improve the synchronization of the neural network.

The simulation time is set to 300 mS, and the correlation coefficient of action potential numerical calculation is collected. The calculation results are shown in Table 4.

**Table 4:** Correlation coefficient of Rabinovich's chemical synaptic coupling network.

**Condition**	***r*(1–25)**	***r*(15–30)**	***r*(20–35)**	***r*(5–45)**
Ring network topology, sine wave stimulation	0.168	0.192	0.167	0.137
Ring network topology, sine wave superposition interference	0.379	0.32	0.218	0.204
NW small-world network topology, sine wave	0.969	0.94	0.922	0.879
NW small-world network topology, sine wave Superimposed interference	0.88	0.856	0.816	0.745

The calculation results show that the NW small-world network has a better effect on the synchronization degree of neurons in the network, whether it is a sine wave stimulation signal or a stimulation signal after the sine wave is superimposed with Gaussian white noise interference. The calculation results of the correlation coefficient and the potential release waveform are mutually corroborated, which can well verify this conclusion. By comparing the correlation coefficient calculation results of the two chemical synaptic coupled NW small-world networks, it can be concluded that the synchronous discharge ability of the Rabinovich synaptic model coupled small-world network is stronger than that of the Hansel chemical synaptic coupled small-world network.

### 3.5 Firing patterns in networks using electrical synaptic coupling model

In addition to a large number of chemical synapses, there are also a certain number of electrical synapses in the human nervous system. Studies have shown that electrical synaptic coupling also plays a non-negligible regulatory role in the nervous system. Therefore, the study of electrical contact is also very important. The synapses of the coupled neural network are changed from chemical synapses to electrical synapses. According to the topology of Figures 3, 4, a network of 100 neurons is constructed in Simulink. The input stimulus signal is selected as the sine wave in Figure 9 and the sine wave is superimposed with Gaussian white noise interference. The simulation time is set to 300 mS, and the synaptic coupling strength is set to 0.1mS/cm^2^. The first neuron in the network is set to receive the stimulation signal applied by the outside world or the stimulation signal after the superposition of interference, and the influence of electrical contact on the synchronous discharge and anti-interference ability of the network is studied. Because the electrical synapse is two-way conduction information, the discharge waveforms of neurons 1, 2, 4, 5, 25, and 50 in the network are selected for comparison.

**Figure 14:**
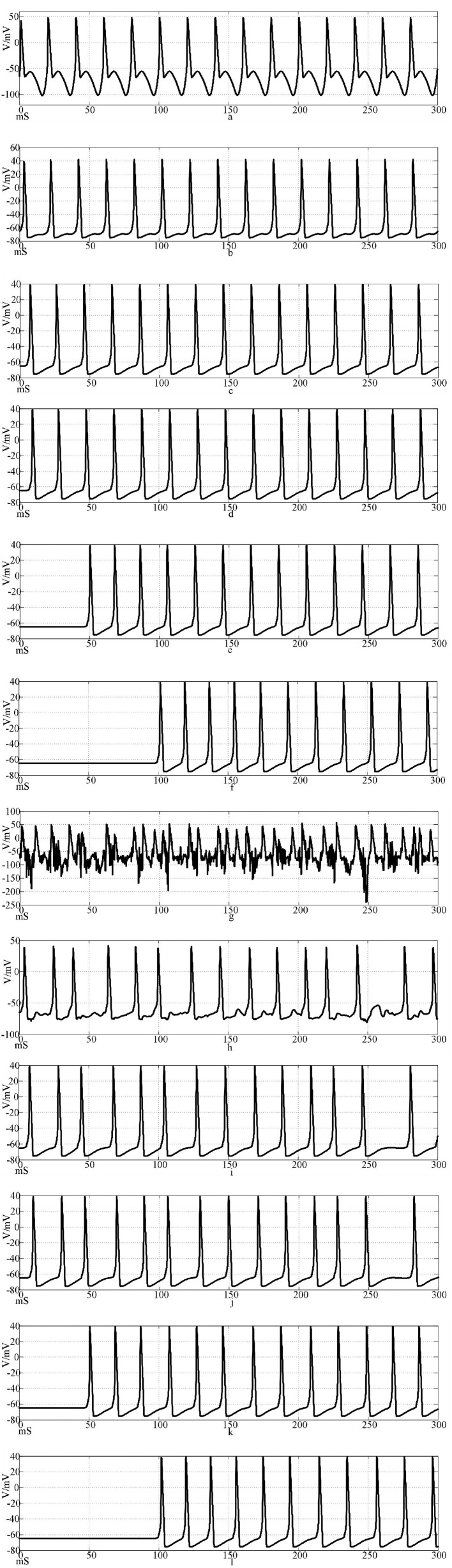
Waveforms of neuronal potentials in a ring network coupled by the electrical synaptic model. [**(a–f)** Action potential waveforms of 1, 2, 4, 5, 25, and 100 neurons without interference were recorded; **(g–l)** The action potential waveforms of neurons 1, 2, 4, 5, 25, and 100 were interfered].

Figures 14a–f shows the discharge waveforms of neurons 1, 2, 4, 5, 25, and 50 in the electrosynaptic coupling loop network after the input of the sine wave stimulation signal. The neurons in the network produce stable action potentials. The action potential firing process of depolarization, reverse polarization, repolarization, and hyperpolarization is complete, reflecting the 'all or none' characteristics. However, except for the first neuron, there is a delay in the firing of other neurons. Figures 14g–l is the discharge waveforms of neurons 1, 2, 4, 5, 25, and 50 after the sinusoidal wave superimposed with Gaussian white noise interference input into the electrical synaptic coupling ring network. After the stimulation signal superimposed interference, the firing of neuron 1 potential in the network produced an obvious subthreshold response and interference. It can be seen from Figures 14b, c that after synaptic transmission, two neurons were greatly reduced by interference, and only a small amount of subthreshold response was produced, while neuron 4 was completely unaffected, and the anti-interference effect was obvious. This result shows that the interference signal can be effectively reduced after synaptic transmission in the electrical synaptic coupling network.

**Figure 15:**
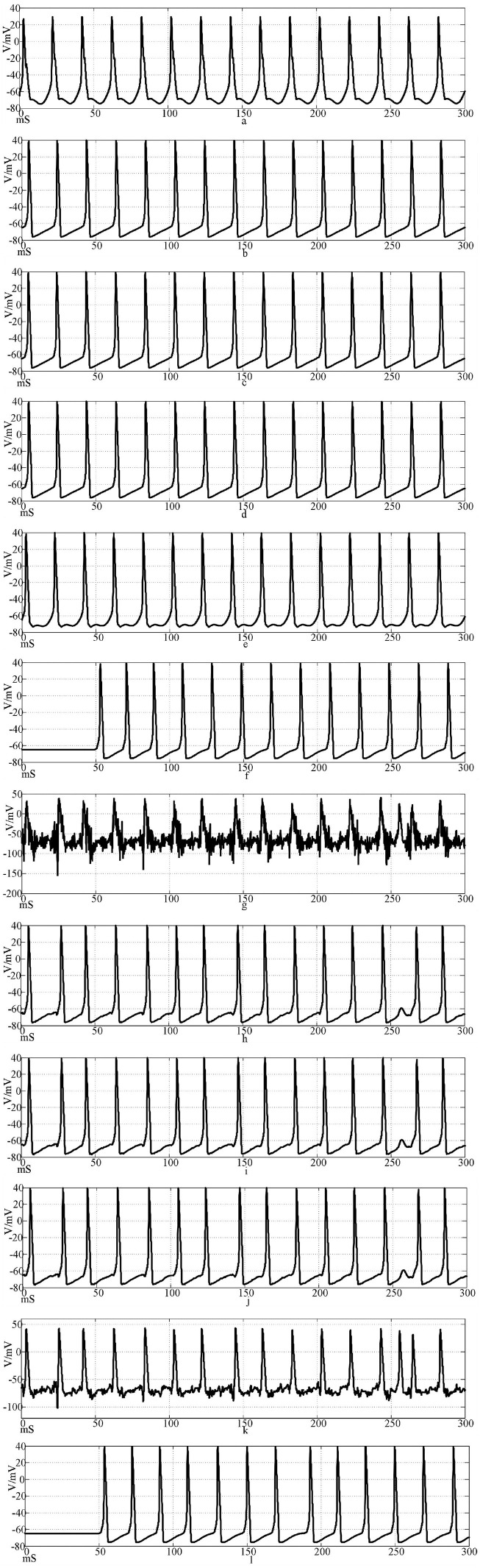
The waveform of neuronal potential in NW small-world network coupled by electrical synaptic model. [**(a–f)** Action potential waveforms of 1, 2, 4, 5, 25, and 100 neurons without interference were recorded; **(g–l)** The action potential waveforms of neurons 1, 2, 4, 5, 25, and 100 were interfered].

Figure 15 shows the action potential waveforms of neurons 1, 2, 4, 5, 25, and 50 in the network after the network topology becomes a more complex NW small-world network. Figures 15a–f is the input of the sine wave stimulation signal. Comparing the waveforms of Figure 14a–f, 15a–f, it is found that when the network topology becomes an NW small-world, in addition to the decrease of the delay of the subsequent neuron potential release and the increase of the degree of synchronous discharge, the neuron action potential release in the network is also more regular and the hyperpolarization process of neuron 1 is weakened. Figures 15g–l is the stimulation signal input of sine wave superimposed with Gaussian white noise interference. Compared with Figures 14g,k, 15g,k waveforms, the first neuron action potential in Fig.15(g) network is relatively less disturbed under the superimposed interference, but the interference of neuron 25 is more serious than that in Figure 14k. When the stimulus information with superimposed interference is input into the ring network, the number of subsequent neuron action potentials decreases, which is improved in the NW small-world network. The overall comparison can be concluded that the delay of subsequent neuron potential release in the small-world network is reduced, and the synchronization degree of action potential release is improved.

The simulation time is set to 300 mS, and the correlation coefficient of action potential numerical calculation is collected. The calculation results are shown in Table 5.

**Table 5:** Correlation coefficient of electrical synaptic coupling network.

**Condition**	***r*(1–25)**	***r*(15–30)**	***r*(20–35)**	***r*(5–45)**
Ring network topology, sine wave stimulation	0.292	0.136	0.134	0.134
Ring network topology, sine wave superposition interference	0.099	0.06	0.016	0.07
NW small-world network topology, sine wave	0.569	0.478	0.31	0.142
NW small-world network topology, sine wave Superimposed interference	0.569	0.309	0.282	0.216

The results of the correlation coefficient calculation show that the NW small-world network is better in the degree of synchronization of neurons in the network, whether it is the sine wave stimulation signal or the stimulation signal after the sine wave superposition Gaussian white noise interference. The calculation results of the correlation coefficient and the potential waveform are mutually corroborated, which can well verify this conclusion. By comparing the correlation coefficient calculation results under the chemical synaptic coupling NW small-world network, it can be concluded that the synchronous discharge ability of the small-world network coupled by the electrical synaptic model is weaker than that of the chemical synaptic coupling small-world network.

By comparing the calculation results of Figures 10, 11, 14, 15, and Formulas (5) and (7) and related parameters, it can be found that the performance of the electrical synaptic coupling network is close to that of the Hansel chemical synaptic coupling network. Hansel chemical synapse is the simplest chemical synaptic model, which is also proved by the experimental results in this section.

## 4 Discussions

The nervous system is a complex information processing system. From mammals to the simplest single-cell organisms, the behavior of organisms depends on the control of the nervous system, and the activities of the nervous system are closely related to bioelectrical signals. There is more or less electromagnetic interference in the environment where organisms live, but organisms can resist interference, which depends on the strong anti-interference ability of neural networks. This anti-interference ability can resist the interference of noise to make the neural network achieve synchronous discharge and improve the accuracy of information processing. In Reference Zhang et al. (2023), the method of constructing spiking neural network to simulate the behavior of the network by IF neuron model was proposed. In Reference Lin et al. (2021), the influence of synaptic coupling strength on the synchronization behavior of neural networks was studied. Reference Ma et al. (2021) studied the robustness mechanism of neuronal information coding of Maeda-Makino hardware neurons and the effects of electromagnetic interference on neuronal cell membrane permeability, sodium channel, potassium channel, and action potential. Reference Zhang et al. (2020) studied the initial value problem of HH model parameters, analyzed its stability, and evaluated the dynamic stability of neurons. Reference Man et al. (2016) studied the degradation phenomenon in the neural network model and proved that with the increase of coupling strength and complexity, the network redundancy decreases. Compared with the current popular synchronization phenomenon and application research of neurons, synapses, and neural networks, this study focuses on revealing the biological source of the anti-interference characteristics of neural networks. The neuronal network is a complex structure coupled by multiple neurons and synapses. The structure that realizes the interconnection and interaction between neurons is synapses. The integration of synapses, neurons, and neural networks can better reveal the source of anti-interference characteristics of neural networks. According to the experimental results of the second part, it can be inferred that the action potential transmission delay has a great influence on the network to achieve anti-interference and synchronous discharge mode. By studying the source of anti-interference mechanism, we can have a deeper understanding of the biological nervous system, reveal the laws of some life phenomena, and promote the development of anti-interference hardware.

In this study, a neural network based on the HH model and chemical synapses was constructed by using the MATLAB platform and numerical simulation method. Through graphical and modular modeling, the topology of the neural network in the biological system is constructed, and the synchronization characteristics and anti-interference ability of the coupled neural network of different synapses are studied. By changing the type of synapses and the topology of neuronal networks, the neuronal networks with different topologies achieve synchronous discharge. The Scope module in Simulink shows the firing of action potentials of different neurons in the network and calculates the correlation coefficient between neurons 1 and 25, neurons 5 and 45, neurons 15 and 30, and neurons 20 and 35 in the neural network. The synchronization and anti-interference effects of different synaptic coupling networks are compared, as well as their effects on the anti-interference effect and synchronization ability of neuronal networks. Comparative simulation found that neurons in the network produced a complete action potential process under external signal stimulation. Different synaptic models coupled networks have different ability to regulate synchronous discharge, and the synchronous discharge state of neurons in the network will change with the change of the topology of the neuron network.

For the neuronal network coupled by the electrical synapse model, the action potential excited by the subsequent neurons transmitted by the same stimulation signal is equal to less than that of the chemical synapse-coupled neuronal network. After the stimulation signal is superimposed with Gaussian white noise interference, the subsequent neurons are conducted multiple times through an electrical synapse to suppress the interference. Under the ring network topology, the electrical contact data generated by sine wave stimulation is relatively stable within four conditions, and the fluctuation range is between 0.134 and 0.292. After superimposing interference signal onto sinusoidal inputs, the correlation coefficient between neurons decreases. Under the NW small-world network topology, the correlation coefficient of action potential increased, and the correlation coefficient of neurons 1 and 25 increased to 0.569. By calculating the correlation coefficient, it can be found that the synchronization degree of neuron discharge in the network after the complex NW small-world topology will also be improved.

As a simple chemical synaptic model, the performance of the Hansel chemical synapse is close to that of electrical synaptic coupling, and the correlation coefficient fluctuates below 0.3. In the case of the NW small-world network, the correlation coefficient increases, which is consistent with the comparison results of electrical contact. However, the anti-interference characteristics are weaker than the NW small-world network coupled by electrical contact, but the anti-interference effect can be achieved. By comparing the correlation coefficient of action potential of four pairs of neurons, it was found that the correlation coefficient between neurons with long distances in the NW small-world network was small. Before and after superposition interference, the correlation coefficients of r(1~25), r(15~30), r(20~35), and r(5~45) were 0.563, 0.539, 0.248, 0.243, and 0.163, 0.096, 0.090, 0.086, respectively. The correlation coefficient decreased significantly after superposition interference.

Rabinovich chemical synaptic model is a complex synaptic model with time delay and considering feedback current. Its comprehensive effect of synchronous discharge ability and anti-interference is the best among the three synaptic models in this study: whether in a ring network or NW small-world network, its subsequent neuron action potential firing delay is the smallest. In the synapse-coupled NW small-world network, the action potential correlation is high. In the case of no superimposed interference: r(5~45) was the lowest, but the correlation coefficient was still 0.879, and the correlation coefficient between neuron 1 and neuron 25 reached 0.969. After superimposed interference, the correlation coefficient range is still between 0.88 and 0.745. The maximum correlation coefficients in the coupling network of electrical synapses and Hansel's chemical synapses were only 0.569 and 0.163. By calculating the correlation coefficient results, the excellent performance of Rabinovich chemical synapses and NW small-world networks can be proved.

The correlation coefficient analysis results are basically the same as the action potential pulse waveform in Figures 10–15. Electrical synapses can achieve two-way conduction of stimulation, so the farthest end of the network is neuron 50, while the farthest end of the chemical synaptic coupling network is neuron 100. The action potential delay of neuron 50 in Figure 14 is 100 mS, and the delay of neuron 100 in Figure 10 is greater than 200 mS. In terms of delay characteristics, due to the bidirectional conduction characteristics, the electrical synapse is better than the Hansel chemical synapse without considering the feedback circuit. Comparing the above two synapses with the complex Rabinovich chemical synapse, the action potential delay of neuron 100 in Figure 12 is less than 50 mS, which is better than the electrical synapse and Hansel chemical synaptic coupling network. It can be seen that the electrical synapse has the characteristics of bidirectional conduction stimulation, which is superior to some simple chemical synaptic models that do not consider delay and feedback current. At the same time, comparing Figures, it can be seen that the action potential delay of neuron 100 in NW small-world network is lower than that in the ring network. Combined with the results of correlation coefficient analysis, it can be explained that the network topology also affects the delay and correlation, and the NW small-world network also enhances the synchronization of the network by reducing the delay. In Figures, the action potential waveform of neuron 1 was distorted after superposition interference. The subliminal response was produced before the interference was not superimposed, which affected the stimulation signal transmission. However, the action potential waveforms of neurons 2, 4, 5, 25, and 100 were restored by the conduction of the neural network, which was close to the waveform before the superposition interference. Based on the analysis of Figures, the following conclusions can be drawn: Different synaptic models have different delays in the coupled network, and the delay affects the synchronization of the network; and the electrical synaptic coupling network is slightly better than the Hansel chemical synaptic coupling network without considering the delay and feedback mechanism due to its bidirectional conduction characteristics, but it is not as good as the Rabinovich chemical synaptic coupling network considering the delay and feedback mechanism. The NW small-world network enhances network synchronization by reducing the conduction delay, and the Rabinovich chemical synapse coupling NW small-world network has the best effect.

It can be seen that synaptic and network conduction delay is one of the important factors affecting the anti-interference ability and synchronous discharge ability of neural networks. In the case of interference, selecting synapses with a time delay mechanism and better performance can improve the synchronization and anti-interference ability of neural networks. At the same time, the complexity of the synaptic conduction structure can also enhance the synchronous discharge and anti-interference ability of the network. In the face of strong interference, the mechanism of the synapse itself and the complexity of the network can be combined to better achieve the effect of network anti-interference. The experimental results in this study have reference significance for the development of anti-interference technology in the fields of neuroscience, computing, and engineering. Future research can focus on exploring the synergy between synaptic mechanisms and network complexity to further enhance the anti-interference performance of neural networks and promote the application of related models in practical anti-interference scenarios.

## 5 Conclusion

The coupling network with different synaptic models will lead to differences in their anti-interference ability and characteristics. The synchronization ability of the neuron network coupled by the electrical synaptic model is generally weaker than that of the neuron network coupled by the chemical synaptic model, and the interference will affect the correlation coefficient results. In the NW small-world network topology, the correlation coefficient is increased compared with the ring network. By comparing the correlation coefficient between the two neurons in the Hansel chemical synaptic coupling network and the electrical synaptic coupling network and the potential waveform of the neurons, it can be seen that the network performance of the two synaptic couplings in the ring network is similar. Comparing the correlation coefficient, the electrical synaptic coupling network in the NW small-world topology has better synchronization ability. However, Hansel chemical synapses have the characteristics of one-way transmission of stimulation signals.

According to the calculation results of the correlation coefficient, the NW small-world network of Rabinovich chemical synaptic coupling has obvious advantages. By comparing the neuron potential waveforms and correlation coefficient calculation results in chapters 2.3, 2.4, and 2.5, it is found that the Rabinovich chemical synapse model shows significant advantages in improving network synchronization and anti-interference characteristics due to its significantly reduced signal transmission delay. The reduction of delay enables the neuron cluster to achieve more accurate phase locking, and the reduction of synchronization error makes the network maintain a stable synchronous discharge mode under noise. At the same time, the NW small-world network constructs more signal transduction pathways than the ring network by establishing redundant connections. This topology optimization shortens the average path length of the network, significantly reduces the conduction delay, improves the synchronization efficiency, and enhances its anti-interference characteristics at the same time. The topology complexity significantly enhances the dynamic robustness of the network through delay optimization.

This study investigates the anti-interference and synchronization dynamics of neuronal networks using the Hodgkin–Huxley (HH) model coupled with electrical synapses (ES), Hansel chemical synapses (HS), or Rabinovich chemical synapses (RS) under two topologies: ring and NW small-world networks. The RS model's feedback mechanism mirrors biological processes that enable precise neural synchronization, offering insights into how real neural circuits balance plasticity and stability. NW small-world topologies align with brain network structural efficiency, suggesting evolutionary optimization for noise resistance. Future research will focus on investigating hybrid networks to mimic brain complexity and identify optimal anti-interference configurations and to explore how spike-timing-dependent plasticity (STDP) or adaptive coupling strengths interact with network dynamics under interference.

## Data Availability

The original contributions presented in the study are included in the article/supplementary material, further inquiries can be directed to the corresponding author/s.
